# Clustered monoallelic mosaicism in twins suggests previously unrecognized path of mutagenesis

**DOI:** 10.1016/j.xhgg.2026.100636

**Published:** 2026-06-23

**Authors:** Jonas Böhnlein, Johann G. Maass, Julia Dennig, Sebastian Burkart, Lilian T. Kaufmann, Michelle Brehm, Kirsten Göbel, Annette Kopp-Schneider, Tim Holland-Letz, Laurine K. Sprehe, Katrin Hinderhofer, Maja Hempel, Christian P. Schaaf

**Affiliations:** 1 Institute of Human Genetics, Heidelberg University Clinic, Heidelberg, Germany; 2 Center for Molecular Biology of Heidelberg University (ZMBH), DKFZ-ZMBH Alliance, Heidelberg, Germany; 3 Division of Genetics and Genomics, Boston Children’s Hospital, Harvard Medical School, Boston, MA, USA; 4 Neuropathology, Institute of Pathology, Heidelberg University Hospital, Heidelberg, Germany; 5 Division Biostatistics, German Cancer Research Center (DKFZ), Heidelberg, Germany; 6 Department of Cardiology, Pulmonology and Angiology, University Hospital Heidelberg, Heidelberg, Germany

**Keywords:** mosaicism, clustered monoallelic mosaicism, cMoMa, genetic nomenclature, Human Genome Variation Society, postzygotic mutation, *HNRNPU*, developmental and epileptic encephalopathy, DNA repair, monozygotic twins, neurodevelopmental disorder

## Abstract

We report monozygotic twins with *HNRNPU*-related neurodevelopmental disorder who harbor two closely spaced mosaic single-nucleotide deletions on the same allele (c.1463del [p.Pro488Glnfs∗13] and c.1466del [p.Lys489Argfs∗12]). The variants are mutually exclusive on individual DNA molecules and result in three distinct cellular lineages within each individual. We term this rare genotypic configuration clustered monoallelic mosaicism (cMoMa). Recognizing the extreme improbability of such a configuration, we systematically explore potential mechanisms for its origin. Based on our analysis, we propose that this genotype arises from a single mutational event in an early embryonic cell, yielding divergent outcomes on sister chromatids. Screening of large datasets (COSMIC and MosaicBase) identified additional cMoMa-like cases, suggesting that the mechanism is not unique to our case but may represent a broader, previously unrecognized path of mutagenesis that extends our current definition of mosaicism.

## Teaser

We describe a previously unrecognized mosaic configuration: two closely spaced mosaic variants on the same allele that never co-occur on any DNA molecule. We term this pattern clustered monoallelic mosaicism (cMoMa) and propose that it arises from a single mutational event rather than two independent events, and we report additional cases.

## Introduction

*HNRNPU* (GenBank: NM_031844.3) encodes the heterogeneous nuclear ribonucleoprotein U, a highly conserved RNA-binding protein involved in RNA splicing, chromatin organization, and nuclear architecture.^1^^,^^2^ Haploinsufficiency of *HNRNPU* is associated with a rare neurodevelopmental disorder (developmental and epileptic encephalopathy 54; MIM: 617391) characterized by developmental delay, moderate to severe intellectual disability, epileptic encephalopathy, and muscular hypotonia.^3^^,^^4^^,^^5^^,^^6^ Additionally, postzygotic mutations in *HNRNPU* have been proposed to cause autism spectrum disorder, indicating that both germline and mosaic variation in this gene can be pathogenic.^7^

In the present study, whole-genome sequencing of monozygotic twins with a clinical phenotype consistent with *HNRNPU*-related neurodevelopmental disorder revealed an unexpected genetic constellation: two closely spaced, mosaic single-nucleotide deletions on the same parental allele, mutually exclusive at the molecular level. Because such a configuration is highly improbable under the conventional assumption of independent mutational events, we systematically interrogated its origin. We assessed two competing models—independent dual mutagenesis versus a single mutational event resolved through divergent sister-chromatid repair—and screened large-scale mosaic and somatic mutation datasets (MosaicBase and COSMIC) for analogous configurations. These analyses lead us to propose clustered monoallelic mosaicism (cMoMa) as a previously unrecognized mosaic configuration, with implications for variant interpretation, recurrence risk estimation, and the broader framework of clustered mutational processes.

## Material and methods

### Whole-genome sequencing and bioinformatics analysis

Libraries were prepared from leukocyte-derived genomic DNA using the NEBNext Ultra II FS DNA PCR-free Library Prep Kit for Illumina, followed by high-throughput sequencing with 150-bp paired-end sequences on the NovaSeq 6000 system (Illumina). The data obtained from sequencing were bioinformatically analyzed using the varfeed pipeline, and the resulting variants were evaluated using the varvis software (v.2.1.0) from Limbus Medical Technologies with a mean coverage of 46. All annotations refer to the human reference genome GRCh38 (hg38). Various prediction programs, some of which are integrated into varvis, were consulted to assess the variants. These include, for example, Fathmm, MetaLr, MetaSvm, MutAssessor, MutTaster, ScSnvAda, ScSnvRf, and SIFT. Information from public databases such as ClinVar, HGMD Professional, OMIM, UCSC, Decipher, and the Database of Genomic Variants (DGV) was also used. The nomenclature of the Human Genome Variation Society (HGVS; http://www.hgvs.org/mutnomen/) is used to classify variants. Pathogenicity is classified based on the American College of Medical Genetics and Genomics and International Agency for Research on Cancer guidelines.^8^^,^^9^

### Sanger sequencing

Genomic DNA extracted from buccal mucosa and from leukocytes of the twins was used for PCR amplification and further sequencing of exon 7 of the *HNRNPU* gene (GenBank: NM_031844.3) comprising both deletions in question (primers available on request). This method has a sensitivity of 97%–99% for identifying sequence alterations in the region of interest.

### Long-read sequencing

Long-read sequencing was performed using the Oxford Nanopore platform. Library preparation was conducted using the Ligation Sequencing Kit V14 (Oxford Nanopore Technologies, SQK-LSK114-XL) according to the manufacturer’s instructions, with modifications based on the Rapid CNS2 approach. In brief, 2.5–3 μg of DNA were sheared to an average fragment length of 25 kb using Covaris g-TUBEs (Covaris, 520079). During the end-prep step, the AMPure XP beads ratio was adjusted to 70 μL. Adapter ligation was performed with 10 min of mixing on a Hula Mixer, and during the final elution step samples were incubated for 3 min at 37°C. Subsequently, 600–700 ng of the prepared library was loaded onto a new MinION Flow Cell R10.4.1 (ONT, FLO-MIN114) and sequenced for 24 h with enabled adaptive sampling that limited data acquisition to sequences of the *HNRNPU* locus ±20 kb. Bioinformatics analysis was carried out using the Rapid-CNS2 pipeline.

### COSMIC analysis

Variants from the COSMIC tumor dataset (229,087 samples; 1,048,574 variants) were filtered to include single-nucleotide deletions only. This restriction was chosen because paired single-nucleotide deletions were the variant configuration observed in the index twins and in the most directly comparable published cases. Variant clusters were defined per sample and gene, and only clusters with inter-variant distances <100 bp were retained (*n* = 59). Prevalence was plotted as a histogram using a sliding window (max_distance = 100 bp; window = 3 bp; step = 2 bp). A kernel density estimate (bandwidth = 2) was overlaid, and a Poisson-based expected distribution (*λ* = 0.8) was added for comparison. All analyses and visualizations were performed in Python.

### MosaicBase analysis

We reanalyzed MosaicBase to identify candidate cMoMa events.^10^ As in the COSMIC analysis, we focused on single-base deletions to match the index configuration and to avoid combining variant classes with distinct mutation rates and interpretative challenges. Entries corresponding to single-cell sequencing datasets (disease: Cockayne syndrome, xeroderma pigmentosum, asymptomatic, human skin fibroblasts, or NA) were excluded, as these studies lacked haplotype resolution required for cMoMa identification. For the remaining entries, we searched for individuals carrying ≥2 distinct variants in the same gene located within ≤10 bp. Redundant or identical calls were collapsed, and distinct variant pairs were reported as cMoMa candidates. Two published haplotype-resolved single-base deletions met these criteria.^11^^,^^12^

### Ethics approval

This small study was approved by the ethics committee of the Medical Faculty Heidelberg (S-632/2023).

## Results

Here, we report monozygotic twin males born at 36 + 1 week of gestation after an uncomplicated pregnancy. Both individuals displayed highly similar clinical features. At birth, they exhibited postaxial hexadactyly of the feet, feeding difficulties, and early failure to thrive. Anthropometric measurements showed short stature and microcephaly. Development was characterized by mild global developmental delay, generalized muscular hypotonia, neurogenic hip dysplasia, and pes planovalgus. Both twins experienced febrile seizures. Behavioral features included reduced risk awareness and limited peer interaction.

**Figure 1:**
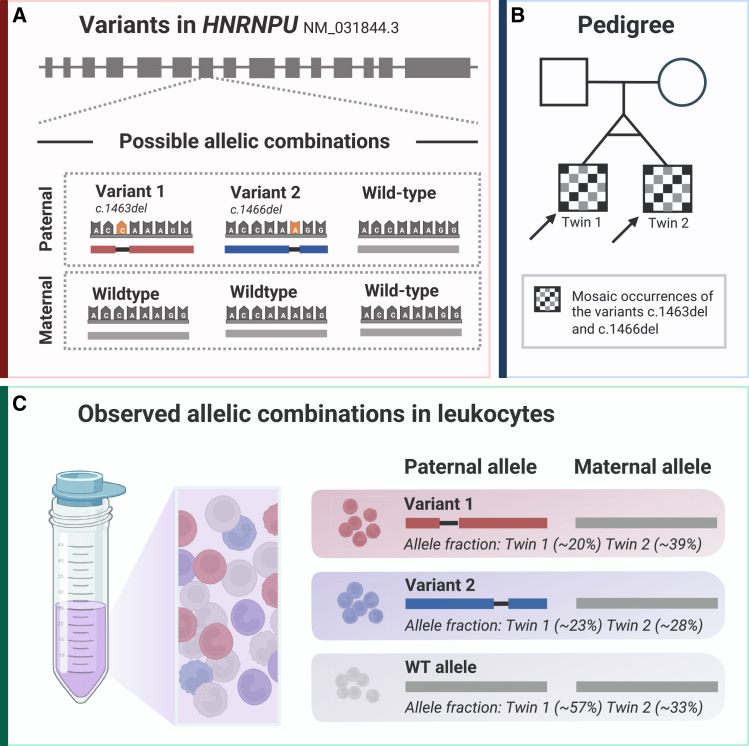
Overview of the genotype discovered in this study (A) Schematic of *HNRNPU* pre-mRNA and the three allelic haplotypes. Two closely spaced 1-bp deletions (c.1463del and c.1466del) occur on the paternal allele, with ambiguous positioning of c.1466del. Long-read phasing confirmed both variants on the paternal haplotype and mutual exclusivity at the molecular level. (B) Pedigree of the family studied. Checkered boxes resemble mosaic occurrences of the variants c.1463del and c.1466del. (C) Illustration of the three different allelic combinations identified in blood leukocytes. Observed allele fractions based on whole-genome sequencing differed between twins, consistent with early postzygotic segregation.

Subsequent whole-genome sequencing (WGS) of leukocyte-derived DNA identified two closely spaced *de novo* 1-bp deletions in *HNRNPU*: c.1463del (p.Pro488Glnfs∗13) and c.1466del (p.Lys489Argfs∗12), each present in mosaic state (Figure 1). Variant-allele fractions (VAFs) ranged from approximately 20% to 39%. Sanger sequencing of DNA from leukocytes and buccal swabs independently confirmed both variants and their mosaic distribution (Figure S1; full details on sequencing methods are provided in supplemental text S1). Combined, the genetic results and clinical phenotype established the diagnosis of *HNRNPU*-related neurodevelopmental disorder.

Notably, the deletions were mutually exclusive at the molecular level: sequencing reads harbored either c.1463del or c.1466del but never both (Figures 1 and S2A). Long-read sequencing further demonstrated that both deletions are located on the paternal allele (Figures 1 and S2B). Together, these findings indicate that each twin harbors three cellular lineages distinguished by the sequence of the paternal allele: one carrying the c.1463del variant, one carrying the c.1466del variant, and one retaining the wild-type sequence, while the maternal allele remains wild-type in all cells. This configuration—two closely spaced *de novo* deletions on the same allele segregating into distinct mosaic lineages and never co-occurring on the same DNA molecule—may represent a previously unrecognized genetic constellation, which we term “clustered monoallelic mosaicism” (cMoMa).

**Figure 2:**
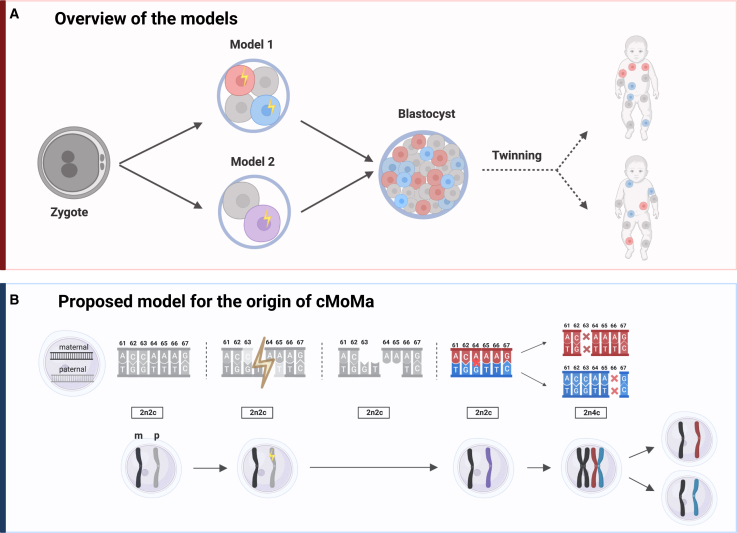
Mechanistic hypotheses for the origin of cMoMa (A) Colored clones indicate lineage segregation through the blastocyst stage and into both twins. (B) Option 2: a singular event. A single mutational event occurs before sister-chromatid formation. Asymmetric processing during repair creates two distinct 1-bp deletions that are subsequently segregated to opposite sister chromatids after DNA replication.

Recognizing cMoMa as a rare genetic constellation, we systematically explored potential mechanisms for its origin. Two models could explain this pattern (Figure 2A).

**Figure 3:**
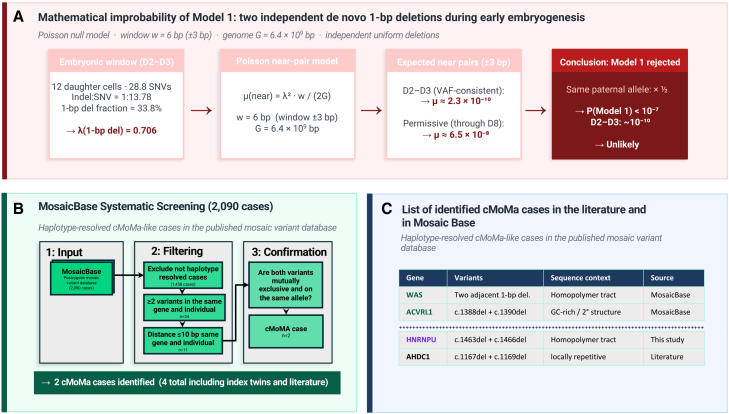
Mathematical calculations and previously identified cMoMa cases (A) Probability calculation for model 1 (two independent *de novo* 1-bp deletions on the same paternal allele during early embryogenesis) using a Poisson near-pair model. Assuming 12 daughter cells, 28.8 SNVs per cell division, an indel/SNV ratio of 1:13.78, and a 1-bp deletion fraction of 33.8%, the expected probability of two independent deletions occurring within ±3 bp on the same allele is ≤2.3 × 10^−10^ (D2–D3 window) and <10^−7^ even under permissive assumptions, effectively rejecting model 1. (B) Systematic screening of MosaicBase (4,647 postzygotic mosaic variant entries) for haplotype-resolved cMoMa-like cases. Filtering for ≥2 variants in the same gene within ≤20 bp on the same phased allele identified two cases; WAS (homopolymer tract) and ACVRL1 (GC-rich secondary structure context). (C) Combined, this gives a total of four reports (five affected individuals) with the specific cMoMa genotype: one from this study (HNRNPU variants), two from MosaicBase (WAS and ACVRL1 variants), and one additional case that we were able to identify in the literature (AHDC1 variants).

Model 1 (null hypothesis) assumes that two independent mutational events produced the two 1-bp deletions (Figure S3A). For the two variants to reside on the same allele yet remain mutually exclusive, the deletions must have arisen in distinct embryonic lineages. Since both twins carry both variants, the events must have preceded twinning and occurred within the first approximately eight embryonic cell divisions.^13^^,^^14^ Quantitative modeling under permissive assumptions for early embryonic mutation rates indicated that the expected occurrence of two independent 1-bp deletions within a ±3 bp window during this period is <10^−7^ (Figure 3A and supplemental text S1).^15^^,^^16^ Restricting the analysis to the second and third embryonic cleavages, which are more consistent with the observed VAFs, the probability decreases further to approximately 10^−10^. Although this calculation is necessarily approximate, as local mutation rates vary across the genome, these estimates indicate that the independent occurrence of two closely spaced deletions during early embryogenesis is unlikely.

Model 2 (alternative hypothesis) assumes that a single mutational event occurred during the earliest embryonic divisions and that subsequent error-prone repair gave rise to divergent sister-chromatid outcomes: a 1-bp deletion on one chromatid and a distinct, nearby 1-bp deletion on the other (Figures 2B and S3B). Following cell division, segregation of the repaired chromatids would produce daughter lineages carrying either variant 1 or variant 2. This mechanism naturally generates a mosaic pattern in which both variants reside on the same parental allele but remain mutually exclusive on individual sequencing reads. It therefore accounts for the molecular exclusivity of the variants, their shared paternal phase, and the mosaic VAFs observed in leukocytes and buccal swabs, including tissue-specific skew. Importantly, this model requires only a single mutational event rather than two independent deletions arising in extremely close proximity.

If cMoMas arise from a reproducible mutational mechanism, similar configurations should occur in other genomic datasets. We therefore explored large-scale mutation resources for comparable patterns, focusing on single-nucleotide deletions. In an exploratory analysis of the COSMIC dataset (229,087 tumor samples), we observed an increased prevalence of closely spaced single-nucleotide deletions compared with a random distribution (Figure S4), suggesting that clustered short deletions occur in large somatic mutation datasets. However, because COSMIC does not provide systematic haplotype-level resolution and cancer variant datasets are influenced by clonal dynamics and variant-calling constraints, these events cannot be assigned to cMoMa. To overcome this limitation, we screened MosaicBase for individuals carrying two distinct variants in the same gene located within 10 bp on the same allele (Figure 3B).^10^ Two published cases met these criteria, each involving haplotype-resolved, mutually exclusive single-base deletions consistent with the cMoMa definition (Table S1).^11^^,^^12^ We acknowledge that, given the small number of haplotype-resolved cases currently available, chance occurrence cannot be formally excluded for the two MosaicBase examples in isolation. In addition, a recently published AHDC1/Xia-Gibbs syndrome case reported two adjacent mosaic deletions, each with allele fractions of approximately 30%–36%, which were mutually exclusive on sequencing reads and assigned to the same parental haplotype by long-read sequencing.^17^ Notably, all four cMoMa descriptions, including the twins described here, consist of pairs of adjacent 1-bp deletions in short repetitive or secondary-structure-prone sequence contexts (Figure 3C). In *HNRNPU* and *WAS*, the deletions occur in short homopolymeric tracts, whereas in *ACVRL1*, they occur within a GC-rich region capable of forming secondary DNA structures. Moreover, in all four instances, the dual-deletion pattern was present across multiple tissues and likely occurred early during development.

## Discussion

We present a case of twins with the newly defined cMoMa genotype, which, combined with previously reported configurations, suggests that cMoMa is not unique to the present case but may represent a recurrent mutational configuration arising through a shared underlying mechanism.

These findings position cMoMa as a mosaic counterpart to other clustered mutational phenomena: in cancer genomes, *kataegis* describes localized hypermutation characterized by clusters of single-nucleotide substitutions; in the germline, multinucleotide mutations capture clusters of closely spaced mutations that occur more often than expected by chance.^16^^,^^18^ Engineered double-strand breaks induced by Cas9 reproducibly yield heterogeneous, sequence-dependent indel spectra at cut sites, often including 1-bp deletions.^19^ Whereas other clustered mutational phenomena originate from episodes involving multiple events, cMoMa is more precisely defined by a single event. It is further characterized by lineage segregation: rather than co-occurrence within the same molecule: divergent outcomes are partitioned across sister chromatids and subsequently across descendant cell lineages, yielding mutually exclusive, monoallelic variants within one individual.

We acknowledge that, given the small number of haplotype-resolved cases currently available, chance occurrence cannot be formally excluded. Further work will be needed to confirm whether our descriptive findings translate into a prevalence of cMoMa that exceeds what would be expected by chance. Future work should aim to characterize the underlying mechanism and determine the true prevalence of cMoMa. Long-read, haplotype-resolved sequencing of trio datasets could enable unbiased detection beyond clinically apparent loci, while single-cell approaches could resolve lineage architecture and developmental timing. Controlled systems such as Cas9-induced breaks at homopolymeric or secondary-structure-prone contexts could directly test whether asymmetric repair reproducibly generates divergent sister-chromatid outcomes.

Discovery of this mechanism might have immediate diagnostic implications. First, it illustrates that mosaicism at a locus does not necessarily reflect expansion of a single allele; instead, multiple distinct alleles may arise from a single event. Second, accurate recognition requires sequencing data with sufficient depth and phasing resolution to detect mutual exclusivity at the molecular level, determine parental origin, and distinguish true clustered variants from complex or recurrent artifacts. Third, for genetic counseling, recurrence risk equals the population baseline. Like other forms of mosaicism, cMoMas arise postzygotically and are therefore incompatible with parental germline mosaicism or inherited transmission. Therefore, in a canonical cMoMa case, future children of the respective parents do *not* have an increased risk of recurrence. This point is particularly counterintuitive in twins: while shared disease in monozygotic twins is usually assumed to reflect inheritance, here both twins are affected by a postzygotic event. Therefore, caution should be exercised to prevent misclassification as germline transmission, as this was the case in a previous cMoMa description.^11^ Conversely, since analogous events occurring prezygotically in the germline would be observed as conventional heterozygous variants, dual mosaic cases may represent one of the few observable manifestations of this mechanism. A second source of underascertainment is genomic context: currently recognized cases derive largely from clinical sequencing, which preferentially detects coding or disease-relevant variants. cMoMa-like events in intronic, intergenic, or otherwise clinically neutral regions would therefore usually remain undetected, suggesting that the true spectrum of this mechanism may be broader than the reported cases indicate.

In summary, we define a previously under-recognized form of mosaicism likely resulting from a single mutational event that generates distinct pathogenic lineages through divergent sister-chromatid repair. We term this configuration “clustered monoallelic mosaicism” (cMoMa), defined by mutually exclusive, same-allele variants that never co-occur on a molecule. By illustrating that a single mutational event may yield multiple lineage-segregated variants, cMoMa challenges the conventional assumption that one mutation produces one variant and expands the current framework for interpreting mosaic and clustered mutational processes.

## Data and code availability

All data supporting the findings of this study are available within the article and its supplemental information. Raw sequencing data (short-read WGS, long-read sequencing, and Sanger traces) are available from the corresponding author upon reasonable request, in accordance with institutional ethics approval and patient consent.

## Acknowledgments

The authors thank Samantha Sarli and Tim Schubert for their intellectual insight and help in revising the manuscript.

## Author contributions

J.B., conceptualization, data curation, formal analysis, investigation, methodology, project administration, visualization, writing – original draft, and writing – review and editing; J.G.M., conceptualization, data curation, formal analysis, investigation, methodology, project administration, visualization, writing – original draft, and writing – review and editing; J.D., conceptualization, data curation, formal analysis, investigation, methodology, project administration, resources, visualization, writing – original draft, and writing – review and editing; S.B., resources and writing – review and editing; L.T.K., formal analysis, investigation, supervision, and writing – review and editing; M.B., investigation and writing – original draft; K.G., investigation and writing – original draft; A.K.-S., formal analysis; T.H.-L., formal analysis; L.K.S., visualization, writing – original draft, and writing – review and editing; K.H., resources, supervision, and writing – review and editing; M.H., writing – review and editing; C.P.S., resources, supervision, and writing – review and editing.

## Declaration of interests

The authors declare no competing interests.

## Declaration of generative AI and AI-assisted technologies in the writing process

During the preparation of this work, the authors used ChatGPT (OpenAI) and Claude (Anthropic) in order to refine the language and improve clarity. After using this tool, the authors reviewed and edited the content as needed and take full responsibility for the content of the published article.

## Web resources

GenBank, https://www.ncbi.nlm.nih.gov/genbank/
OMIM, https://www.omim.org/

## Supplemental information

Document S1. Supplemental text, Figures S1–S4, and Table S1

Document S2. Article plus supplemental information

## References

[1] Kiledjian M., Dreyfuss G. (1992). Primary structure and binding activity of the hnRNP U protein: binding RNA through RGG box. Embo j.

[2] Nozawa R.-S., Boteva L., Soares D.C., Naughton C., Dun A.R., Buckle A., Ramsahoye B., Bruton P.C., Saleeb R.S., Arnedo M. (2017). SAF-A Regulates Interphase Chromosome Structure through Oligomerization with Chromatin-Associated RNAs. Cell.

[3] Hamdan F.F., Srour M., Capo-Chichi J.-M., Daoud H., Nassif C., Patry L., Massicotte C., Ambalavanan A., Spiegelman D., Diallo O. (2014). De Novo Mutations in Moderate or Severe Intellectual Disability. PLoS Genet..

[4] Carvill G.L., Heavin S.B., Yendle S.C., McMahon J.M., O'Roak B.J., Cook J., Khan A., Dorschner M.O., Weaver M., Calvert S. (2013). Targeted resequencing in epileptic encephalopathies identifies de novo mutations in CHD2 and SYNGAP1. Nat. Genet..

[5] Thierry G., Bénéteau C., Pichon O., Flori E., Isidor B., Popelard F., Delrue M.-A., Duboscq-Bidot L., Thuresson A.-C., van Bon B.W.M. (2012). Molecular characterization of 1q44 microdeletion in 11 patients reveals three candidate genes for intellectual disability and seizures. Am. J. Med. Genet..

[6] Need A.C., Shashi V., Hitomi Y., Schoch K., Shianna K.V., McDonald M.T., Meisler M.H., Goldstein D.B. (2012). Clinical application of exome sequencing in undiagnosed genetic conditions. J. Med. Genet..

[7] Lim E.T., Uddin M., De Rubeis S., Chan Y., Kamumbu A.S., Zhang X., D'Gama A.M., Kim S.N., Hill R.S., Goldberg A.P. (2017). Rates, distribution and implications of postzygotic mosaic mutations in autism spectrum disorder. Nat. Neurosci..

[8] Richards S., Aziz N., Bale S., Bick D., Das S., Gastier-Foster J., Grody W.W., Hegde M., Lyon E., Spector E. (2015). Standards and guidelines for the interpretation of sequence variants: a joint consensus recommendation of the American College of Medical Genetics and Genomics and the Association for Molecular Pathology. Genet. Med..

[9] Plon S.E., Eccles D.M., Easton D., Foulkes W.D., Genuardi M., Greenblatt M.S., Hogervorst F.B.L., Hoogerbrugge N., Spurdle A.B., Tavtigian S.V. (2008). Sequence variant classification and reporting: recommendations for improving the interpretation of cancer susceptibility genetic test results. Hum. Mutat..

[10] Yang X., Yang C., Zheng X., Xiong L., Tao Y., Wang M., Ye A.Y., Wu Q., Dou Y., Luo J. (2020). MosaicBase: A Knowledgebase of Postzygotic Mosaic Variants in Noncancer Disease-related and Healthy Human Individuals. Genom. Proteom. Bioinform..

[11] Dobbs A.K., Yang T., Farmer D.M., Howard V., Conley M.E. (2007). A possible bichromatid mutation in a male gamete giving rise to a female mosaic for two different mutations in the X-linked gene WAS. Clin. Genet..

[12] Eyries M., Coulet F., Girerd B., Montani D., Humbert M., Lacombe P., Chinet T., Gouya L., Roume J., Axford M.M. (2012). ACVRL1 germinal mosaic with two mutant alleles in hereditary hemorrhagic telangiectasia associated with pulmonary arterial hypertension. Clin. Genet..

[13] Hardy K., Handyside A.H., Winston R.M. (1989). The human blastocyst: cell number, death and allocation during late preimplantation development in vitro. Development.

[14] Scott L. (2002). The origin of monozygotic twinning. Reprod. Biomed. Online.

[15] Spencer Chapman M., Ranzoni A.M., Myers B., Williams N., Coorens T.H.H., Mitchell E., Butler T., Dawson K.J., Hooks Y., Moore L. (2021). Lineage tracing of human development through somatic mutations. Nature.

[16] Besenbacher S., Sulem P., Helgason A., Helgason H., Kristjansson H., Jonasdottir A., Jonasdottir A., Magnusson O.T., Thorsteinsdottir U., Masson G. (2016). Multi-nucleotide de novo Mutations in Humans. PLoS Genet..

[17] Hu J., Dawood M., Mehta H.H., Pasham D., Kaur M., Kalra D., Walker K., Gingras M.-C., Lupski J.R., Sabo A. (2026). Double Mosaicism in Xia-Gibbs Syndrome. Am. J. Med. Genet..

[18] Alexandrov L.B., Nik-Zainal S., Wedge D.C., Aparicio S.A.J.R., Behjati S., Biankin A.V., Bignell G.R., Bolli N., Borg A., Børresen-Dale A.-L. (2013). Signatures of mutational processes in human cancer. Nature.

[19] Allen F., Crepaldi L., Alsinet C., Strong A.J., Kleshchevnikov V., De Angeli P., Páleníková P., Khodak A., Kiselev V., Kosicki M. (2018). Predicting the mutations generated by repair of Cas9-induced double-strand breaks. Nat. Biotechnol..

