# Supplementary material for: Clustered monoallelic mosaicism in twins suggests previously unrecognized path of mutagenesis
Source: HGG Adv. 2026 Jun 23;7(4):100636. doi: 10.1016/j.xhgg.2026.100636 (PMC13356607; doi:10.1016/j.xhgg.2026.100636)
Supplement: Document S1. Supplemental text, Figures S1–S4, and Table S1 [file mmc1.pdf]

HGGA, Volume 7

Supplemental information

Clustered monoallelic mosaicism in twins suggests previously unrecognized path of mutagenesis

Jonas Böhnlein, Johann G. Maass, Julia Dennig, Sebastian Burkart, Lilian T. Kaufmann, Michelle Brehm, Kirsten Göbel, Annette Kopp-Schneider, Tim Holland-Letz, Laurine K. Sprehe, Katrin Hinderhofer, Maja Hempel, and Christian P. Schaaf

S1: Probability calculations for cMoMa genotype..2

Figure S1: Sanger sequencing of leukocyte-derived and buccal-derived DNA 5

Figure S2: Whole-genome sequencing of the leukocyte-derived DNA............................6

Figure S3: Proposed molecular mechanism underlying cMoMa formation. 7

Figure S4: Analysis of the COSMIC Mutant Census dataset..8

Table S1: Overview of the cMoMa cases presented in this study.....................................9

1 A null model for near pairs under Poisson event counts

We model the number N of early 1-bp deletions as Poisson with mean λ. Conditional on N = n, the n deletion coordinates are assumed to be independent and uniformly random over the diploid genome {1, . . . , G}.[1] We call two events a near pair if their coordinates differ by at most w/2 bases, i.e. they lie within a symmetric window of total length w around an index site. Here we set w = 6 bp to represent the window ±3 bp excluding the index base (distinct positions).

General result (used throughout). For N ∼ Poisson(λ) and w ≪ G, the expected number of near pairs is derived as follows.

Given N = n deletions, there are n(n − 1)/2 possible pairs, and each pair has probability about w/G of being within the same local window. Therefore,

$$\mathbb{E}[\#\text{near pairs} \mid N = n] = \frac{n(n-1)}{2} \cdot \frac{w}{G}.$$

Taking the expectation over N gives

$$\mathbb{E}[\#\text{near pairs}] = \mathbb{E}\left[\frac{N(N-1)}{2} \cdot \frac{w}{G}\right] = \frac{w}{2G}\,\mathbb{E}[N(N-1)].$$

For any random variable, $\mathbb{E}[N(N-1)] = \mathbb{E}[N^2] - \mathbb{E}[N]$. Using $\text{Var}(N) = \mathbb{E}[N^2] - (\mathbb{E}[N])^2$, we can write

$$\mathbb{E}[N(N-1)] = \text{Var}(N) + (\mathbb{E}[N])^2 - \mathbb{E}[N].$$

For a Poisson variable, $\text{Var}(N) = \mathbb{E}[N] = \lambda$, so

$$\mathbb{E}[N(N-1)] = \lambda + \lambda^2 - \lambda = \lambda^2.$$

Hence,

$$\boxed{\mu_{\text{near}} = \frac{\lambda^2}{2} \cdot \frac{w}{G}.}$$

This is the expected number of near-pair events under the null model of independent, uniformly distributed deletions.

---

[1]This simplified null model assumes that each deletion arises independently and with equal probability across the diploid genome, ignoring local sequence-context biases. It thus provides a baseline estimate of how rare such events would be under independence.

2 Early scenario restricted to divisions 2 and 3 (2→4 and 4→8 cells)

Rationale. A first-cleavage (zygote) event would eliminate the wild-type paternal lineage, which is inconsistent with the observed VAFs; therefore we restrict the early scenario to the second and third cleavages.

Assumptions. Diploid genome size G ≈ 6.4 × 109 bp and window w = 6 bp. For the first three cleavages, Chapman et al. estimate ≈ 2.4 SNVs per daughter cell per division. We convert SNVs to 1-bp deletions (1-bp dels) using an indel:SNV ratio of 1:13.78 and a 1-bp deletion fraction among indels of 33.8%.

Expected number of 1-bp deletions and near pairs

Divisions 2 and 3 produce 4 + 8 = 12 daughter cells, giving

$$S_{(2,3)} = 12 \times 2.4 = 28.8 \text{ SNVs}.$$

Converting to 1-bp deletions yields the Poisson mean

$$\lambda_{(2,3)} = 28.8 \times \frac{1}{13.78} \times 0.338 = 28.8 \times 0.07257 \times 0.338 \approx \mathbf{0.7064}.$$

Thus

$$\mu_{\text{near}} = \frac{\lambda^2_{(2,3)}}{2} \cdot \frac{w}{G} = \frac{0.7064^2}{2} \cdot \frac{6}{6.4 \times 10^9} \approx \mathbf{2.34 \times 10^{-10}}.$$

3 Aggregating through the 8th mitosis (twinning window)

Assumptions. We aggregate across the first eight mitotic divisions (to ∼ 256 cells). For divisions 1–3 we use 2.4 SNVs per daughter per division; for divisions 4–8, we use < 0.9 SNVs per daughter per division. The same indel:SNV and 1-bp deletion fractions apply.

Expected number of 1-bp deletions and near pairs

Divisions 1–3: 2 + 4 + 8 = 14 daughters ⇒ 14 × 2.4 = 33.6 SNVs.

Divisions 4–8: 16 + 32 + 64 + 128 + 256 = 496 daughters ⇒ 496 × 0.9 ≈ 446.4 SNVs.

Total: S ≈ 33.6 + 446.4 = 480 SNVs.

Convert to 1-bp deletions:

$$\lambda_{(\leq 8)} = 480 \times \frac{1}{13.78} \times 0.338 \approx 480 \times 0.07257 \times 0.338 \approx \mathbf{11.8}.$$

Hence

$$\mu_{\text{near}} = \frac{\lambda_{(\le 8)}^2}{2} \cdot \frac{w}{G} = \frac{11.8^2}{2} \cdot \frac{6}{6.4 \times 10^9} \approx \mathbf{6.53 \times 10^{-8}}.$$

Interpretation. In a simplified model that assumes deletions occur independently, are uniformly distributed across the diploid genome, and follow Poisson-distributed event counts, the expected number of near-pair deletions within ±3 bp is exceedingly small. When considering only the second and third embryonic cleavages, this expectation is on the order of 10−10. Even when extending the calculation to include all events through the eighth mitosis, the expected near-pair count rises only to about 10−8. These estimates indicate that two closely spaced 1-bp deletions are highly unlikely to result from independent mutational events under this model.

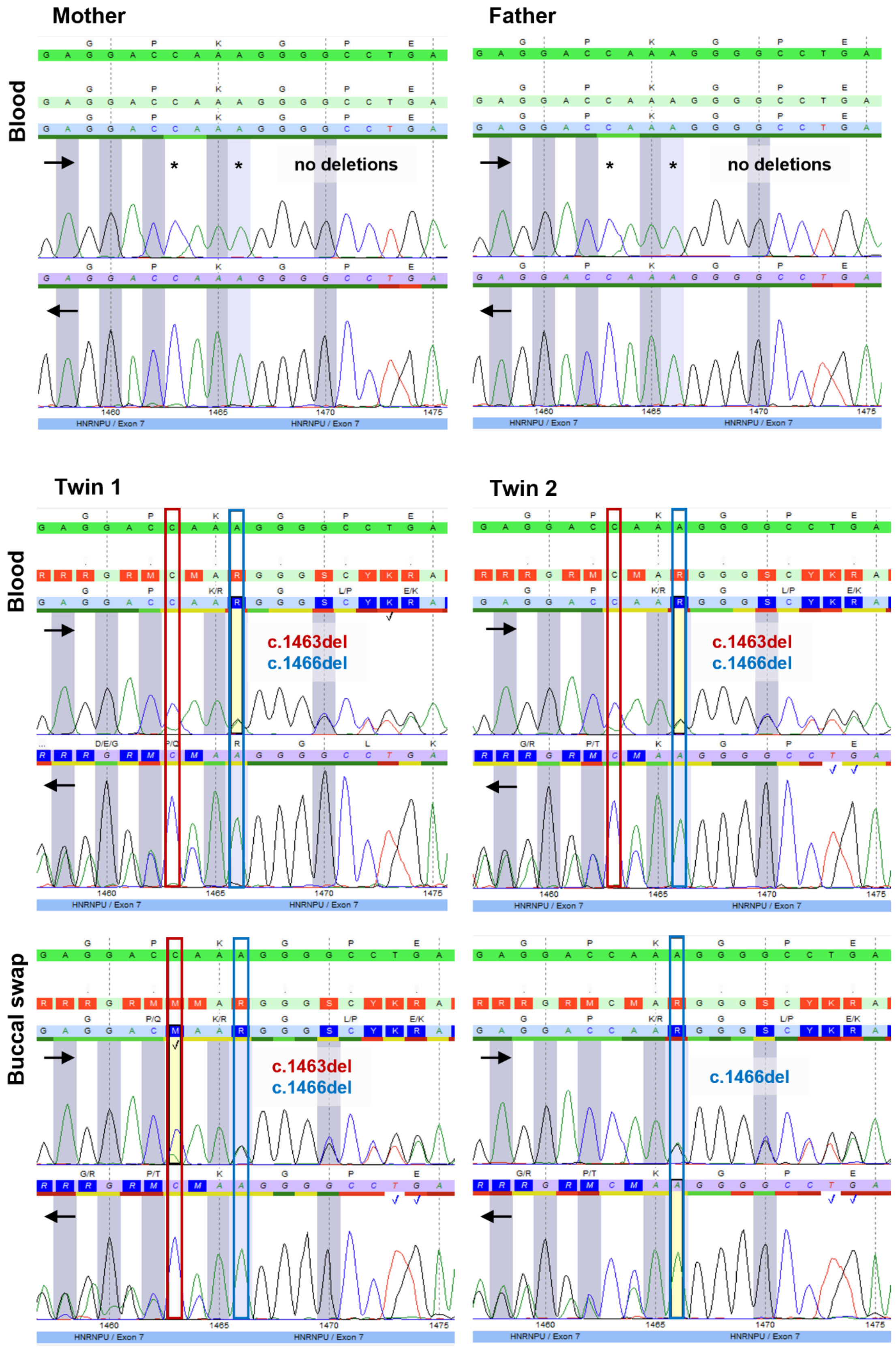

Figure S1: Sanger sequencing of leukocyte-derived and buccal-derived DNA

In twin 1, both variants (*HNRNPU* NM_031844.3; c.1463del and c.1466del) were present in mosaic form in both tissues. In contrast, twin 2 exhibited both deletions in mosaic form only in leukocyte-derived DNA, while only one heterozygous deletion (c.1466del) was detected in the buccal swab, consistent with lineage skew and assay sensitivity limits.

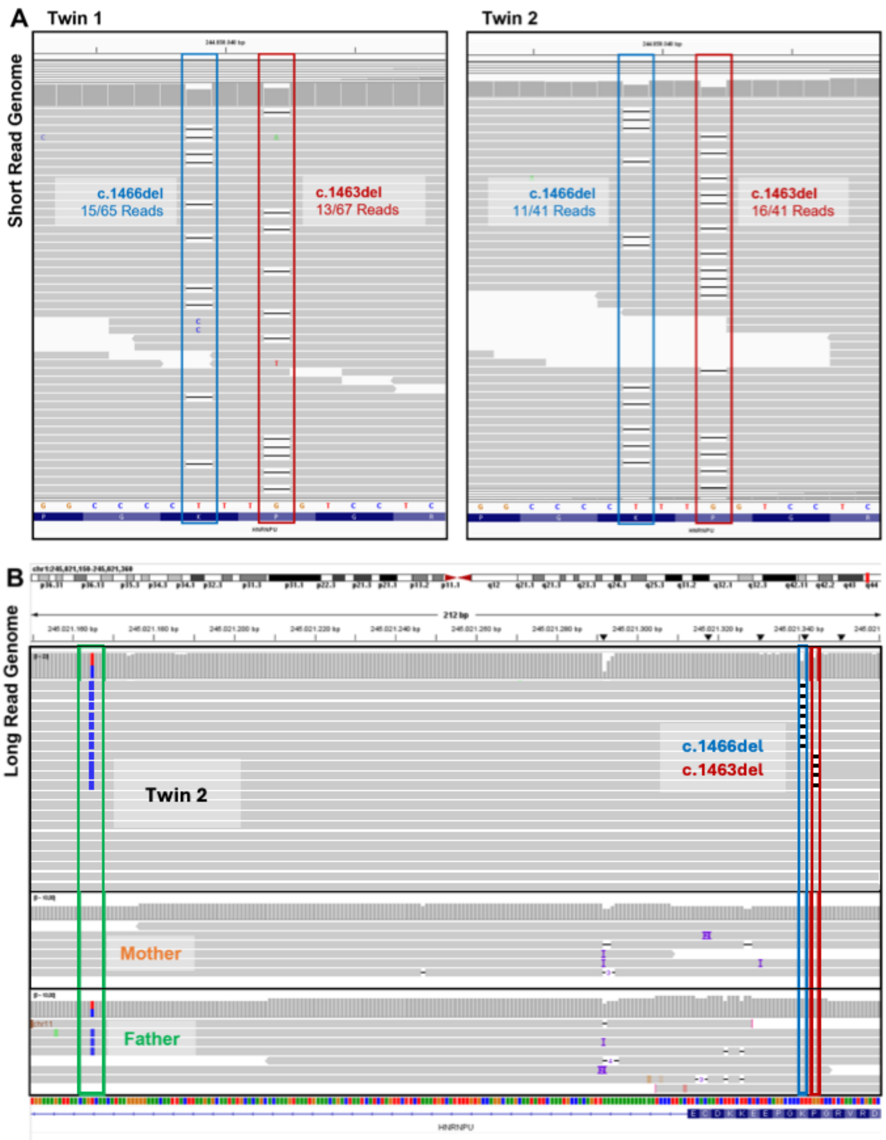

Figure S2: Whole-genome sequencing of the leukocyte-derived DNA

(A) Short-read whole-genome sequencing of leukocyte-derived DNA from the twins and their parents identified two closely spaced *de novo* 1-bp deletions in *HNRNPU* (NM_031844.3; c.1463del and c.1466del) on chromosome 1. They were present in mosaic state with variant allele frequencies (VAFs) ranging from ~20-39%. Short-read genome sequencing data demonstrated that both deletions were never observed on the same read.

(B) Nanopore long-read sequencing was used to determine the allelic phase, revealing both on the paternal allele, indicated by a paternal SNP (green box) on the same read as both deletions (blue/red box). For Twin 2, reads are displayed separated by haplotype to more clearly illustrate the three distinct genotypes across haplotypes (re-arranged in powerpoint); the underlying sequencing data are identical to the original submission.

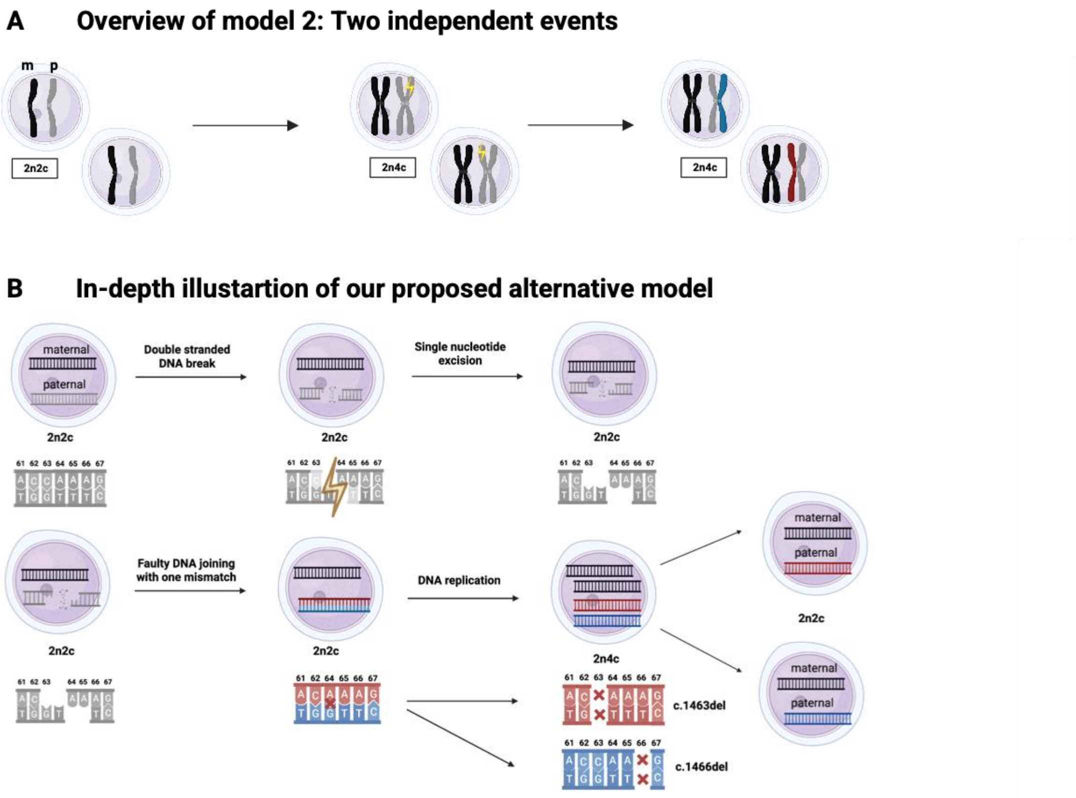

Figure S3: Proposed molecular mechanism underlying cMoMa formation.

(A) Illustration of model 2. Two independent events. Two distinct de novo 1-bp deletions arise on the paternal allele in different early embryonic lineages. Because the events occur in separate cells, no double-mutant molecules are produced. Subsequent cleavages propagate two mutually exclusive mosaic lineages (red, blue)

(B) Schematic representation of a potential mechanism of cMoMa formation: A DSB arises on the paternal allele, followed by end processing and single-nucleotide loss on each break end. Re-ligation of the processed fragments introduces a transient base-pair mismatch, producing two subtly different sister chromatids. After replication, the divergent chromatids segregate into separate daughter cells, yielding two independent mosaic lineages that together recapitulate the genotype observed in this study.

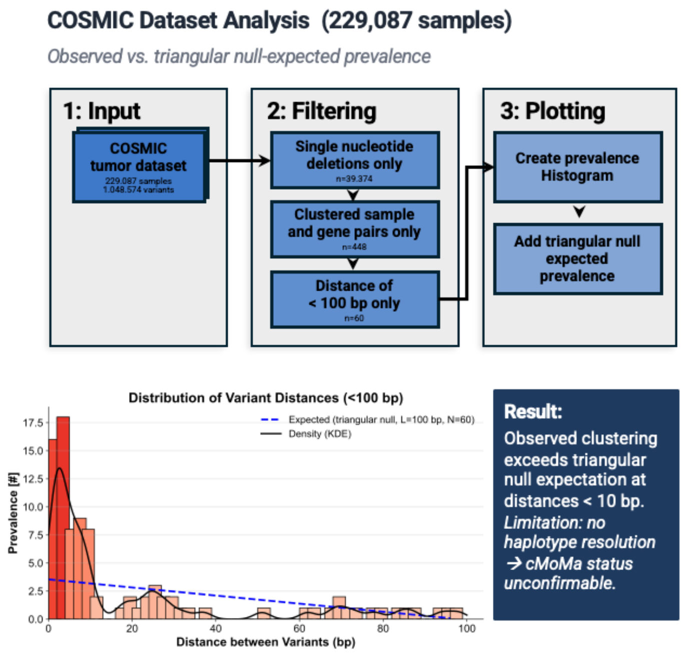

Figure S4: Analysis of the COSMIC Mutant Census dataset

Analysis of the COSMIC Mutant Census dataset (229,087 tumor samples; 1,048,575 variants) for clustered single-nucleotide deletions. After filtering for sample–gene pairs with ≥2 unique 1-bp deletions (448 pairs; 371 samples), inter-variant genomic distances were plotted as a sliding-window histogram. Observed variant clustering at distances <10 bp markedly exceeds the triangular null expectation (L=100 bp, N=60), supporting non-random co-occurrence of proximal deletions in cancer. Haplotype resolution was not available in this dataset, precluding definitive cMoMa classification.

Table S1: Overview of the cMoMa cases presented in this study

	This study	Eyries et al., 2012	Dobbs et al., 2007	Hu et al., 2026
**Gene Name**	HNRNPU	ACVRL1	WAS	AHDC1
**Variant 1 (HGVS)**	c.1463del	c.1388del (p.Gly463Alafs*2); within G(2) tract	c.755del (within C(3) tract c.755–c.757; codon 241)	c.1167delG
**Variant 2 (HGVS)**	c.1464del (within A(3) tract c.1464–c.1466)	c.1390del (p.Leu464*); within C(2) tract	c.758del (within C/A homopolymer; codon 242)	c.1169delC
**Distance (bp)**	1–3 bp	1–4 bp	1–3 bp	1–2 bp
**Sequence Context**	Located within an A/T-rich repetitive region	Local hairpin structure	Short run of cytosines and a brief palindromic motif	Locally repetitive GGCC sequence at mutation site
**Variant type**	1-bp deletions	1-bp deletions	1-bp deletions	1-bp deletions
**Affected individual**	Monozygotic twins	Single female with HHT + PAH	Single carrier female (II:2) for WAS	Single 10-year-old female with Xia-Gibbs syndrome
**Evidence for same haplotype / mutual exclusion**	Long-read phasing shows mutually exclusive deletions on the same paternal allele	Microsatellite haplotyping; same maternal haplotype	Allele-specific PCR: both deletions on same great-grandpaternal haplotype	Long-read WGS and amplicon sequencing showed mutually exclusive deletions.
**Notes**	Detected in blood and buccal DNA; no double-mutant reads; early embryonic origin	Early embryonic origin	Authors correctly identified the dual-deletion genotype but misinterpreted the origin as “bichromatid mutation in a male gamete”; the mosaic distribution indicates a cMoMa event. Germline mosaicism, since each variant was passed on.	Detected in blood and buccal DNA; variant fractions summed to approximately 50%; authors propose likely origin in the zygote with resolution into two daughter products at the first cell division
